# Effects of Cassava−Chili Intercropping at Different Planting Densities on Soil Bacterial Community Structure

**DOI:** 10.3390/microorganisms14071560

**Published:** 2026-07-16

**Authors:** Fei Liang, Shuangjiang Li, Jun Wang, Haihong Xie, Mo Chen, Hao Sheng, Yong Song

**Affiliations:** 1 College of Horticulture, Hunan Agricultural University, Changsha 410128, China; liangfei2026@163.com (F.L.); arceso@163.com (S.L.); wj1923535553@163.com (J.W.); 18175996412@163.com (H.X.); 13805684311@163.com (M.C.); 2 Yuelushan Laboratory, Changsha 401128, China; 3 College of Resources, Hunan Agricultural University, Changsha 410128, China; 4 Hunan Provincial Potato Engineering and Technology Research Center, Changsha 410128, China

**Keywords:** intercropping, cassava, chili, soil biochemical properties, soil bacterial communities

## Abstract

This study established three intercropping densities of cassava and chili (chili plant spacing of 0.4 m, 0.5 m, and 0.6 m, denoted as IA, IB, and IC, respectively), using cassava monoculture (MC) and chili monoculture (MP) as controls, to systematically investigate differences in soil biochemical properties and soil bacterial community structure under different cassava−chili cropping systems. The results showed that soil pH, catalase activity, and acid phosphatase activity were higher in the IB and IC treatments than in the monoculture treatments, while alkali-hydrolyzable nitrogen (AN), available phosphorus (AP), and available potassium (AK) contents were lower in the IA and IC treatments compared to the monoculture treatments. Alpha diversity was higher in the intercropping systems than in the monoculture systems, and the soil bacterial communities exhibited greater richness and diversity. In the intercropping systems, the relative abundances of Verrucomicrobiota, Gemmatimonadota, and the common dominant soil genus *Candidatus_Udaeobacter* increased, while those of Actinobacteriota, Proteobacteria, and the genera *Acidothermus* and *Chujaibacter* decreased. Redundancy analysis indicated that the soil bacterial community was significantly influenced by soil catalase activity and total nitrogen. In summary, cassava−chili intercropping enhances soil nutrient levels and enzyme activity while reshaping the structure of the soil bacterial community.

## 1. Introduction

Cassava (*Manihot esculenta* Crantz), as the world’s sixth-largest food crop, is widely used in the food and chemical industries due to the high starch content of its tubers, and it offers multiple applications as a specialty grain, an energy source, and livestock feed [1,2]. Due to limited cultivation area and historically low land utilization rates, China’s domestic total production is low, making it highly dependent on imports; it has now become the world’s largest importer of cassava [3]. Chilis (*Capsicum annuum* L.) are widely cultivated around the world as both a vegetable and a seasoning. They are rich in various nutrients, such as vitamin C and carotenoids, and possess beneficial pharmacological effects, including antioxidant, anti-inflammatory, and anticancer properties. As public awareness of the nutritional value of chilis continues to grow, consumer demand for them continues to rise [4,5]. Cassava has a relatively long growing season and grows slowly in the early stages, resulting in low resource utilization efficiency. In contrast, chilis have a short growing season and compact plants. Intercropping the two crops allows for complementarity in their spatial ecological niches and increases the overall yield of the system [6,7]. Therefore, exploring suitable cassava chili intercropping models holds significant practical importance for alleviating the cassava supply gap, meeting chili demand, and improving land use efficiency.

Previous studies have shown that, compared to monoculture, intercropping systems offer significant advantages in terms of increasing crop yield and improving crop quality [8,9,10]. For example, intercropping winter lupin with triticale enhances weed control; although this reduces the grain yield of lupin, it increases the total yield of the system [11]. Intercropping wheat with fava beans reduced the individual yields of both wheat and fava beans, but the land-equivalent ratio of the intercropping system was greater than 1, and the total system yield exceeded that of monoculture [12]. Intercropping loquat, bayberry, and citrus with tea plants increased the free amino acid content of tea leaves, which is beneficial for the formation of green tea quality [13]. Intercropping *Platycodon grandiflorus* with chilis significantly improved the intrinsic quality of *Platycodon grandiflorus*, markedly increasing its protein and total saponin content, demonstrating a clear intercropping advantage [14]. Furthermore, through niche complementarity and synergistic resource utilization among species, intercropping systems exert multidimensional effects on soil physical structure, chemical fertility, and microbial communities [15,16]. The mulberry alfalfa intercropping system significantly increased soil available nutrients and total carbon content, while also enhancing the richness and diversity of bacterial communities [17]. Cassava-peanut intercropping significantly increased soil alkali-hydrolyzable nitrogen, available potassium, pH, and soil urease activity, and increased the number of rhizosphere microorganisms, thereby improving soil quality [18]. Intercropping cassava with soybeans alters the accumulation patterns of metabolites and the structure of microbial communities in continuous-cropping soils, thereby alleviating continuous-cropping problems such as soil acidification, compaction, and nutrient depletion [19]. Intercropping cassava with maize significantly increased soil pH and available potassium content, and enhanced the abundance of the Proteobacteria and Actinobacteria phyla [20]. Appropriate cassava intercropping systems can effectively enhance land productivity and soil health, as well as improve soil physical and chemical properties and promote microbial activity [21].

In summary, intercropping systems have demonstrated significant effects in enhancing crop yield and quality, improving soil quality, and increasing nutrient use efficiency. Cassava has a long growing season and slow early growth, resulting in low resource utilization efficiency, whereas chilis have a short growing season and compact plants. Intercropping these two crops can achieve complementary spatial niches and increase the overall yield of the system. Existing research on cassava intercropping has primarily focused on leguminous crops, with very few systematic studies on cassava chili systems, which combine high nutritional and economic value. Studies have shown that while cassava chili intercropping reduces chili yield per unit area, it significantly increases the system’s total economic benefit. Furthermore, proper regulation of intercropping density is key to balancing interspecific competition and achieving increased yields and efficiency [22]. Soil bacteria are the most active and diverse microbial group in soil ecosystems, playing an irreplaceable role in organic matter decomposition, nutrient transformation, carbon and nitrogen cycles, and the maintenance of soil structure [23]. Previous studies have shown that the composition and function of bacterial communities directly influence soil nutrient availability and crop nutrient use efficiency [24]. In intercropping systems, the intertwined root systems of different crops—with variations in the types and quantities of secretions they produce—can shape a unique rhizosphere microenvironment, which in turn regulates the diversity and functional composition of soil bacterial communities [25]. Therefore, elucidating changes in the structure of soil bacterial communities within intercropping systems and their relationship with soil physicochemical properties and enzyme activity is of great significance for understanding the ecological regulatory mechanisms of intercropping systems. To this end, this study used cassava and chili as experimental materials and established a gradient of intercropping densities to systematically investigate the effects of this intercropping system on the biochemical properties of the soil and bacterial communities, with the aim of revealing its potential agricultural benefits and ecological regulation capabilities. The hypotheses of this study are: (1) Different intercropping densities can alter soil biochemical properties; (2) Different intercropping densities can cause significant changes in the richness and composition of soil bacterial communities; (3) Optimal intercropping density is associated with soil biochemical properties and soil bacterial communities.

## 2. Materials and Methods

### 2.1. Test Materials

This experimental study was conducted from January 2022 to April 2026 at the Xiangyan Seed Industry Experimental Base in Chunhua Town, Changsha County, Changsha City, Hunan Province, China (28.3106° N, 113.2974° E), and at Hunan Agricultural University. The cassava variety tested was NanZhi 199, provided by the Wuming Agricultural Technology Extension Station in Guangxi (Nanning, China), and the chili variety was Xiangyan No. 55, bred by Hunan Xiangyan Seed Industry Co., Ltd. (Changsha, China). The basic physicochemical properties of the experimental site were as follows: pH 4.70, organic matter 20.6 g·kg^−1^, alkali-hydrolyzable nitrogen 97 mg·kg^−1^, available phosphorus 20.9 mg·kg^−1^, available potassium 109 mg·kg^−1^, total nitrogen 1.24 g·kg^−1^, total phosphorus 0.51 g·kg^−1^, total potassium 11.9 g·kg^−1^. Fertilizer application rates: 100 kg of NPK compound fertilizer (15-15-15), 50 kg of urea (46.4%), 50 kg of potassium sulfate (52%), and 25 kg of calcium magnesium phosphate fertilizer (12%). Pest and disease control: Apply a 600-fold dilution of 72% mancozeb anganese zinc wettable powder once 30 days after chili transplanting and again at the onset of flowering to control late blight; upon detection of aphids or tobacco cutworms, apply a 2000-fold dilution of 10% imidacloprid wettable powder via targeted spraying. Weed management: Cultivation and weeding were performed three times: 15 days and 45 days after chili transplanting, and before cassava plants closed the rows. Irrigation management: During the trial period, irrigation was managed based on field moisture content (approximately 25%) and crop water requirements. Climatic conditions: From January to November 2022, the average temperature at the experimental site was 18.3 °C. During the chili growing season (January–July), the average temperature was 19.1 °C, while during the cassava growing season (April–November), it was 24.6 °C.

**Figure 1:**
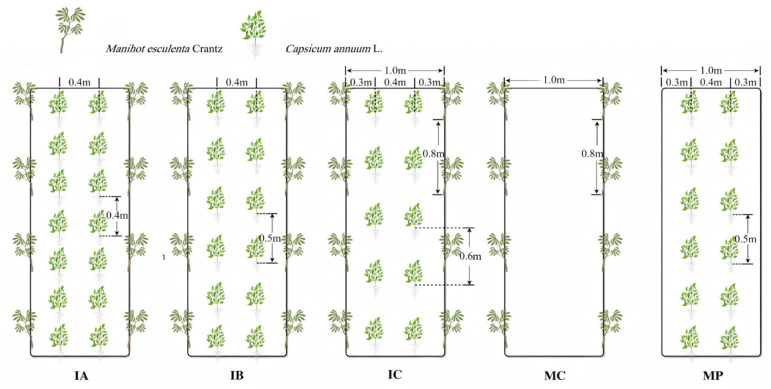
Schematic diagram of planting. Each plot uses ridge cultivation, with ridges 1.0 m wide, furrows 0.6 m wide, and ridges 0.3 m high. The area of each plot is 1.6 × 13 m = 20.8 m^2^. IA, chili plants spaced 0.4 m apart; IB, chili plants spaced 0.5 m apart; IC, chili plants spaced 0.6 m apart; MC, cassava monoculture; MP, chili monoculture.

A single-factor randomized block design was employed. The experiment comprised five treatments: three different densities of cassava–chili intercropping, namely IA (chili plant spacing of 0.4 m), IB (chili plant spacing of 0.5 m), and IC (intercropped chili plant spacing of 0.6 m), with pure cassava (MC) and pure chili (MP) serving as controls. Each treatment was replicated three times, resulting in a total of 15 plots. The planting pattern consisted of two rows of chilis interplanted between cassava rows (as shown in Figure 1). Chili seedlings were sown on 21 January 2022, and transplanted on April 9; cassava cuttings were planted on the same day, with one plant per hole, and missing plants were promptly replaced. Finally, chilis were harvested in batches from 28 May to 11 July 2022, with a total growth period of 171 days; cassava was harvested on 20 November 2022, with a total growth period of 223 days, of which 93 days were spent in symbiosis with chilis.

### 2.2. Measurement of Crop Yield and Quality

Chilis: When the chilis reached maturity, we used an electronic scale to weigh the fruits on each plant. Vitamin C and reducing sugar contents were measured using a test kit provided by Suzhou Grace Biotechnology Co., Ltd. (Suzhou, China).

Cassava: At the time of cassava tuber maturity, 30 plants were randomly selected from each treatment, and the weight of each individual tuber was measured using an electronic scale. The starch and reducing sugar contents were determined using a test kit provided by Suzhou Grace Biotechnology Co., Ltd.

### 2.3. Collection of Soil Samples

Soil samples were collected in November (during the cassava tuber−ripening period) using the five-point sampling method, which involves setting up five sampling points—four corner points and one central point—within the sampling area. Soil samples were collected from each of these points and then mixed to form a composite sample representative of the average conditions across the entire area [26]. For intercropping treatments, soil samples were collected from a vertical depth of 0–20 cm at the midpoint between cassava and chili plants; for cassava and chili monocropping treatments, soil samples were collected from a vertical depth of 0–20 cm at the midpoint between adjacent plants. After sampling, the soil samples were thoroughly mixed. A portion was stored in a −80 °C freezer for soil DNA extraction and high-throughput sequencing, while another portion was air-dried, ground using a 40-mesh sieve, and sieved for use in analyzing the soil’s physicochemical properties and enzymatic activity. All analytical results were calculated based on the weight of the air-dried soil.

### 2.4. Determination of Soil Physicochemical Properties

Soil pH: Place 10 g of soil sample in a 50 mL tall beaker and add 25 mL of deionized water (1:2.5, weight/volume). Stir well, let stand for 30 min, calibrate the pH meter using a standard solution, insert the electrode into the suspension, and record the reading once it has stabilized. Soil organic matter (SOM): Add 0.8 mol/L potassium dichromate and concentrated sulfuric acid to 0.5 g of soil sample. After boiling and cooling, add o-phenanthroline indicator and titrate with 0.2 mol/L ferrous sulfate. Alkaline-hydrolyzable nitrogen (AN): Place 2 g of soil sample in the outer chamber of a diffusion dish; add 2 mL of boric acid indicator to the inner chamber and 1.0 mol/L sodium hydroxide to the outer chamber. Incubate at a constant temperature of 40 °C for 24 h. After removal, titrate the absorbate in the inner chamber with 0.005 mol/L hydrochloric acid. Soil available phosphorus (AP): Add 0.5 mol/L sodium bicarbonate solution to 5 g of soil sample, shake and extract for 30 min, filter, and determine the filtrate using a molybdenum–antimony colorimetric reagent. Available potassium (AK) in soil: Add ammonium acetate solution to 5 g of soil sample, shake for 30 min at 150–180 rpm, filter, and determine directly using a flame photometer. Total nitrogen (TN) in soil: Add 1.8 g of mixed catalyst and 5 mL of concentrated sulfuric acid to 0.5 g of soil sample. After digestion in a digestion furnace at 420 °C, alkalize and distill the sample using a Kjeldahl apparatus. Titrate the distillate with a 0.02 mol/L hydrochloric acid standard solution. Total phosphorus (TP) in soil: Add sodium hydroxide to 0.2 g of soil sample and fuse in a muffle furnace at 640 °C for 15 min. After cooling, dissolve in water, filter, and add a molybdenum–antimony color-inhibiting agent to the filtrate. Determine the concentration via spectrophotometry at 700 nm. Total potassium (TK) in soil: Following the same method as for total phosphorus (alkali fusion, dissolution, and dilution to volume), directly analyze the filtrate for potassium content using a flame photometer [27].

### 2.5. Determination of Soil Enzyme Activity

Four enzyme activities were measured using kits provided by Suzhou Grace Biotechnology Co., Ltd. S-UE (soil urease activity): Add urea and citrate buffer to 0.1 g of soil sample. Incubate at 37 °C for 24 h, and then add sodium phenolate and sodium hypochlorite solution. Allow to develop color at 37 °C for 20 min, and measure the absorbance at a wavelength of 578 nm [28]. S-SC (soil sucrase activity): Add to 0.1 g of soil sample toluene, sucrose, and acetic acid buffer. Incubate at 37 °C for 4 h, and then centrifuge and collect the supernatant. Add 3,5-dinitrosalicylic acid reagent for color development, and measure the absorbance at a wavelength of 540 nm [29]. S-CAT (soil catalase activity): Add distilled water and hydrogen peroxide to 0.1 g of soil sample. Incubate with shaking at 25 °C for 30 min, and then add a color development probe reagent. React at 25 °C for 10 min. Centrifuge and collect the supernatant; add a color development reagent and measure the absorbance at a wavelength of 510 nm [30]. S-ACP (soil acid phosphatase activity): Add to 0.1 g of soil sample toluene and p-nitroaniline phosphate. After shaking at 37 °C for 1 h, centrifuge and collect the supernatant. Measure the absorbance at a wavelength of 405 nm [31].

### 2.6. DNA Extraction, PCR Amplification, and High-Throughput Sequencing

Total soil DNA was extracted using the HiPure Soil DNA Mini Kit, and DNA concentration and integrity were assessed using NanoDrop, Qubit, and agarose gel electrophoresis. The bacterial 16S rRNA gene V3–V4 region was amplified using primers 341F (5′-CCTACGGGNGGCWGCAG-3′) and 805R (5′-GACTACHVGGGTATCTAATCC-3′). The PCR reaction was performed in a 50 μL system under the following conditions: 5 min pre-denaturation at 95 °C; 30 cycles of 1 min denaturation at 95 °C, 1 min annealing at 60 °C, and 1 min extension at 72 °C; followed by a final 7 min extension at 72 °C. After purification, the amplification products were used to construct sequencing libraries, which were then sequenced using paired-end sequencing on the Illumina NovaSeq 6000 platform. After separating the raw sequencing data by barcode, quality filtering was performed using FASTP v0.18.0. FLASH (v1.2.11) was used to assemble paired-end reads into tags (minimum overlap of 10 bp; mismatch rate ≤ 2%), and low-quality tags were further filtered (truncated at bases with consecutive low-quality scores ≤ 3, retaining sequences with a run of high-quality bases ≥ 75% of the tag length). Subsequently, DADA2 (v1.14.1) was used to remove primer sequences, construct an error model for noise reduction, and remove chimeric sequences (using the UCHIME algorithm) to obtain ASVs (amplicon sequence variants). To avoid the influence of differences in sequencing depth between samples on subsequent diversity analysis, the ASV abundance tables for all samples were rarefied and normalized to the minimum number of sequences per sample. Species classification and annotation were performed using the RDP Classifier v2.2 software based on the SILVA database (version 138.2). The raw 16S rRNA gene sequencing data have been deposited in the NCBI Sequence Read Archive (SRA) under BioProject accession number PRJNA1475226.

### 2.7. Statistical Analysis

Data were organized and processed using Microsoft Excel 2016. The normality of the data was assessed using the Shapiro–Wilk test, and homogeneity of variances was verified using Levene’s test. After confirming that the data met the assumptions of normality and homogeneity of variances, a one-way analysis of variance (ANOVA) was performed using SPSS 26.0, followed by Duncan’s multiple range test for multiple comparisons (*p* < 0.05). Use Origin 2024 and the BenagenCloud platform to plot and analyze data. Alpha diversity indices were calculated using QIIME2 based on ASV tables, and one-way ANOVA and Duncan’s multiple range test were used to assess the significance of differences in alpha diversity indices among treatments (*p* < 0.05); for β-diversity analysis, principal coordinate analysis (PCoA) was performed based on the Bray–Curtis distance matrix. The distance matrix was calculated using the vegan package in R, and PERMANOVA was used to test for significant differences in bacterial community structure among treatments; redundancy analysis (RDA) was conducted using the vegan package in R, and Monte Carlo permutation tests were employed to assess the significance of environmental factors and RDA axes.

## 3. Results

### 3.1. Effects of Intercropping on Crop Yield and Quality

As shown in Table 1, there were significant differences in cassava and chili yields between the intercropping treatments and the monoculture treatment. For cassava, the yield order across treatments was IC > MC > IB > IA. The low-density intercropping (IC) treatment yielded significantly more than the monoculture (MC) treatment (*p* < 0.05), with a 13.98% increase compared to MC. Meanwhile, the medium-density intercropping (IB) and high-density intercropping (IA) treatments showed no significant difference compared to the monoculture (MC) treatment (*p* > 0.05). For chilis, the yield order across treatments was MP > IB > IC > IA. The intercropping treatments (IA and IC) yielded significantly less than the monoculture (MP) treatment (*p* < 0.05), with reductions of 12.54% and 11.65%, respectively, compared to MP; however, there was no significant difference between the medium-density intercropping (IB) and the monoculture (MP) treatments (*p* > 0.05). In terms of total yield, all intercropping treatments were significantly higher than the cassava monoculture (MC) and chili monoculture (MP) (*p* < 0.05). Economic benefits were calculated based on the 2023 average market prices of cassava and chilis in China. The results indicated that the economic benefits of all intercropping treatments were significantly higher than those of the monoculture treatments (*p* < 0.05), with the low-density intercropping (IC) treatment yielding the highest total economic benefit.

As shown in Table 2, the starch and reducing sugar contents of cassava in all intercropping treatments were significantly higher than those in the monoculture (MC) treatment (*p* < 0.05), increasing by 8.25–19.33% and 19.87–76.69%, respectively, compared to MC. Significant differences were observed among the intercropping treatments (*p* < 0.05), with the low-density intercropping (IC) treatment having the highest starch content and the high-density intercropping (IA) treatment having the highest reducing sugar content. For chilis, the Vc content in intercropping treatments IB and IC showed no significant difference compared to the monoculture MP (*p* > 0.05), while IA was significantly lower than the other treatments; regarding reducing sugar content, the monoculture MP was significantly lower than the medium-density intercropping treatment (IB), but significantly higher than the high-density IA treatment (*p* < 0.05), with no significant difference from the low-density intercropping treatment (IC) (*p* > 0.05). Among these, the low-density intercropping treatment IC yielded the best overall quality for chilis, while the quality of the high-density intercropping treatment (IA) was somewhat lower compared to the monoculture MP.

### 3.2. Effects of Intercropping on Soil Physicochemical Properties

**Figure 2:**
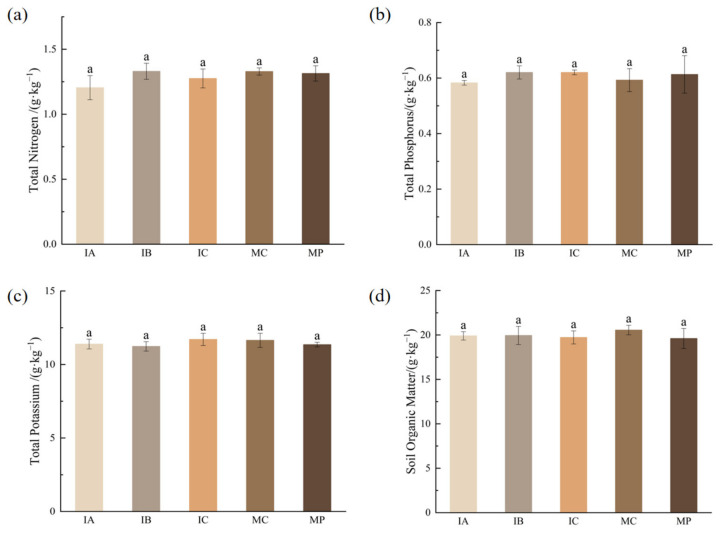

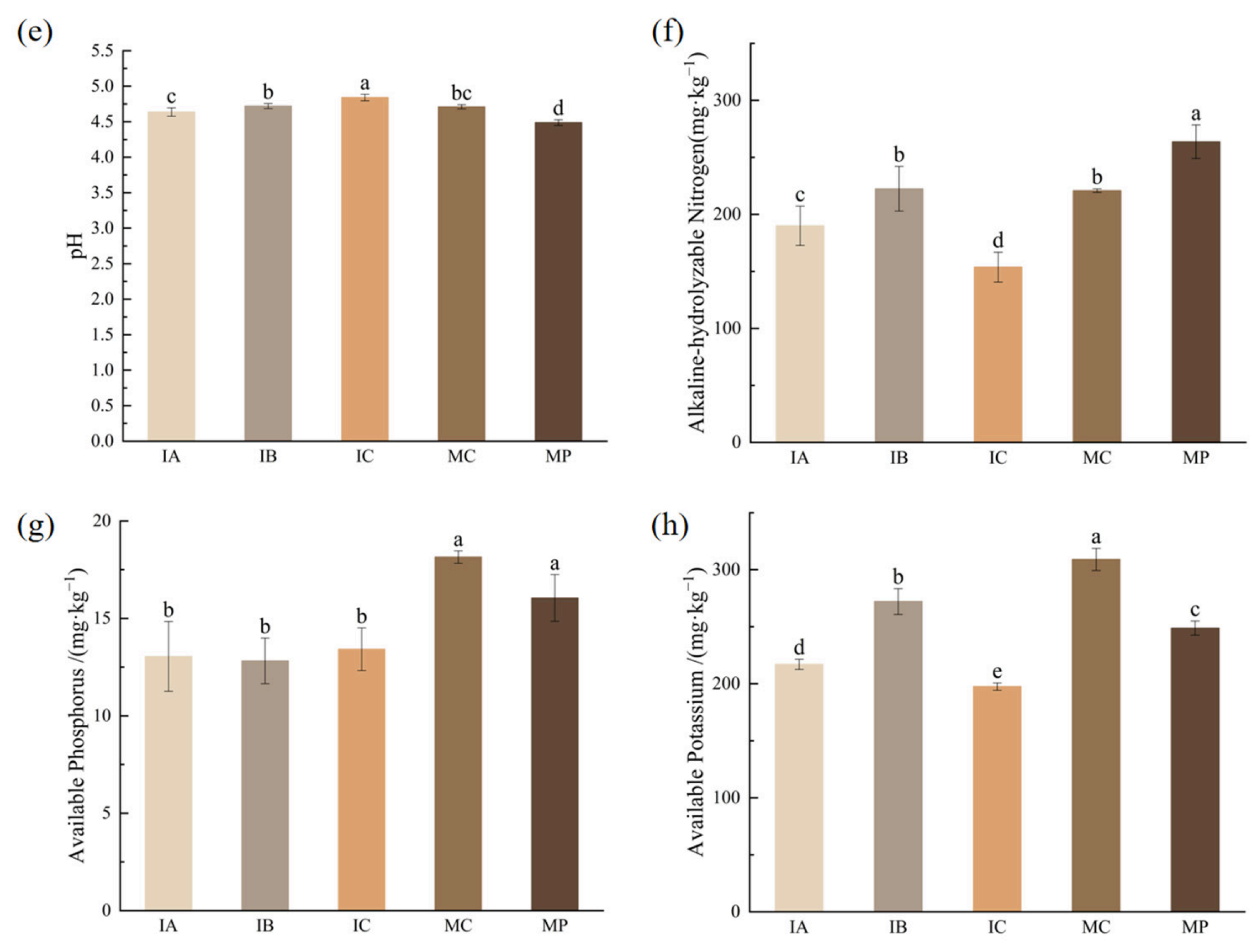
Soil physical and chemical properties. Different letters denote significant differences (*p* < 0.05). (**a**) The effect of intercropping on total nitrogen. (**b**) The effect of intercropping on total phosphorus. (**c**) The effect of intercropping on total potassium. (**d**) The effects of intercropping on soil organic matter. (**e**) The effect of intercropping on pH. (**f**) The effect of intercropping on alkaline-hydrolyzed nitrogen. (**g**) The effect of intercropping on available phosphorus. (**h**) The effect of intercropping on available potassium.

As shown in Figure 2, compared with monoculture, there were no significant changes in soil organic matter, total nitrogen, total phosphorus, and total potassium content under any intercropping treatment, whereas soil pH and available nutrient content were significantly affected. Specifically, the pH values across the different treatments were as follows: IC > IB > MC > IA > MP. Compared with the MP monoculture, the pH values in the IC, IB, and IA treatments increased significantly by 7.80%, 5.12%, and 3.34%, respectively, while the pH value in the MC cassava monoculture was significantly lower than that in the IC treatment (*p* < 0.05), but showed no significant difference compared with the IA and IB treatments (*p* > 0.05). In terms of alkali-hydrolyzable nitrogen content, compared with the MP monoculture, the IA, IB, and IC treatments reduced the content by 27.97%, 15.64%, and 41.72%, respectively; compared with the MC monoculture, the IA and IC treatments reduced the content by 14.01% and 30.42%, respectively (*p* < 0.05), while there was no significant difference between the IB and MC treatments (*p* > 0.05). The available potassium content showed the following order: MC > IB > MP > IA > IC. MC was significantly higher than all intercropping treatments, and MP was significantly higher than IA and IC, but significantly lower than the IB treatment; significant differences were observed among all intercropping treatments (*p* < 0.05). Compared with the MC treatment, the intercropping treatment showed a significant reduction in available phosphorus content of 26.02% to 29.33%; compared with the MP treatment, it showed a significant reduction of 16.39% to 20.12% (*p* < 0.05). In summary, the trends in soil nutrient content varied under different intercropping densities, indicating that intercropping density has a selective effect on the regulation of soil chemical properties.

### 3.3. Effect of Intercropping on Soil Enzyme Activity

**Figure 3:**
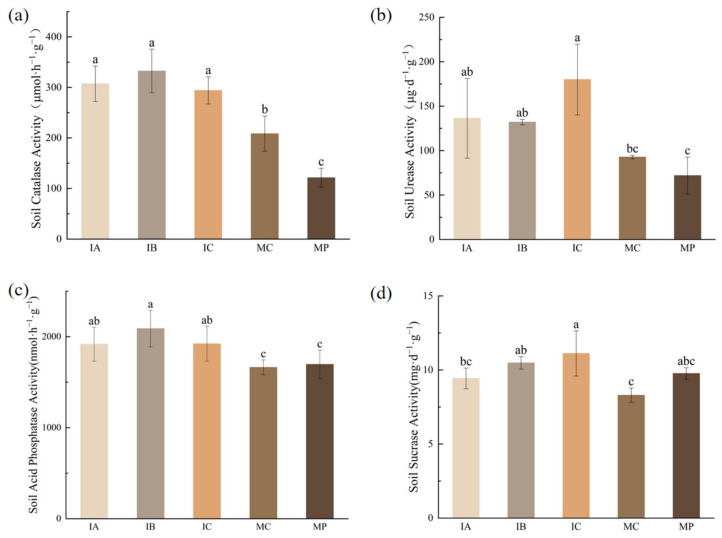
Soil enzyme activity. Different letters denote significant differences (*p* < 0.05). (**a**) The effect of intercropping on soil catalase activity. (**b**) The effect of intercropping on soil urease activity. (**c**) The effect of intercropping on soil acid phosphatase. (**d**) The effect of intercropping on soil sucrase.

As shown in Figure 3, the soil catalase and acid phosphatase activities in all intercropping treatments were significantly higher than those in the cassava monoculture (MC) and chili monoculture (MP) treatments. Specifically, soil catalase activity increased by 41.06–59.52% and 142.35–174.06% compared to MC and MP, respectively, and acid phosphatase activity increased by 15.32–25.62% and 18.08–23.19% compared to MC and MP, respectively; however, no significant differences were observed among the intercropping treatments. Soil urease activity followed a trend of IC > IA > IB > MC > MP across treatments; MC was significantly lower than IC but showed no significant difference from IA and IB; MP was significantly lower than all intercropping treatments. Soil sucrase activity followed the trend of IC > IB > MP > IA > MC; MC was significantly lower than all intercropping treatments, while MP showed no significant differences compared to the other intercropping treatments. In summary, compared to monoculture, intercropping generally increased soil enzyme activity, which is beneficial for improving the soil’s biochemical environment and enhancing nutrient transformation capacity.

### 3.4. Effects of Intercropping on Soil Bacterial Community Structure

#### 3.4.1. Amplicon Sequencing Data

**Figure 4:**
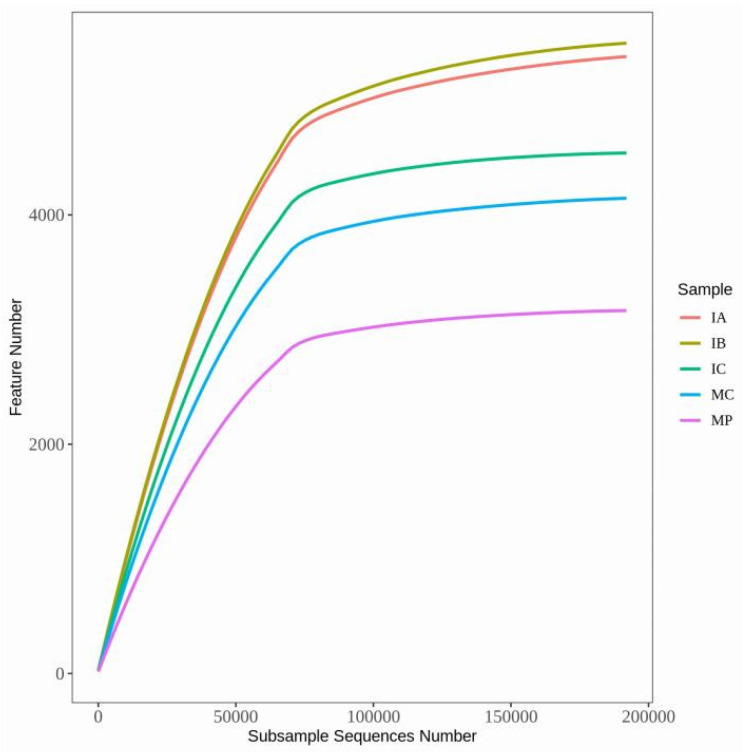
Dilution curve.

The V3–V4 regions of the 16S rRNA gene were sequenced for 15 samples from the intercropping and monocropping treatments. After barcode assembly, a total of 5,121,067 paired reads were obtained, with each sample yielding at least 266,348 paired reads and an average of 341,404 paired reads. After quality control filtering, a total of 4,828,438 high-quality sequences were obtained as input for the DADA2 denoising workflow, with a quality control retention rate of 94.3%. Following further filtering, denoising, assembly, and chimera removal using DADA2, a total of 3,857,910 non-chimeric sequences were obtained. The final number of valid sequences per sample ranged from 199,850 to 334,344, with an overall sequence retention rate of 79.9%. The dilution curves for each sample leveled off after approximately 8000 sequences were extracted (Figure 4), indicating that the sequencing depth was adequate to cover the major taxonomic groups of the soil bacterial community. After DADA2 denoising, a total of 78,560 ASVs were identified across all samples, with the number of ASVs per sample ranging from 3009 to 5522.

#### 3.4.2. Effects of Intercropping on the Composition of Bacterial Phyla and Genera

**Figure 5:**
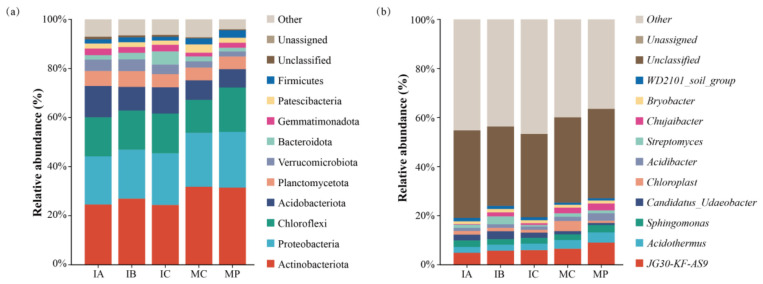
Species composition of soil bacterial communities. (**a**) Species composition of soil bacterial communities at the phylum level. (**b**) Species composition of soil bacterial communities at the genus level.

As shown in Figure 5 and Table 3, at the phylum level, the top 10 soil bacterial communities by relative abundance across all treatments included Actinobacteriota, Proteobacteria, Chloroflexi, Acidobacteriota, Planctomycetota, Verrucomicrobiota, Patescibacteria, Bacteroidota, Gemmatimonadota, and Firmicutes. Compared with the monoculture treatments, MC and MP, the intercropping treatments significantly increased the relative abundances of Acidobacteriota and Verrucomicrobiota. Specifically, the abundance of Acidobacteriota increased by 21.46–60.98% and 28.78–70.68% compared with MC and MP, respectively, with the most significant increase observed in the high-density intercropping treatment (IA). The abundance of Verrucomicrobiota increased by 54.15–88.93% and 86.60–128.71% compared to MC and MP, respectively, with the most significant increase observed in the medium-density intercropping treatment (IB). The relative abundance of Planctomycetota in all intercropping treatments also increased to varying degrees compared with MC and MP, with increases ranging from 2.30% to 23.61% and from 3.29% to 24.81%, respectively, with the most significant increase observed in IB (*p* < 0.05). In contrast, intercropping treatments significantly reduced the relative abundances of Actinobacteriota and Proteobacteria compared to monocropping, with decreases of 15.22–23.36% and 14.30–22.54% compared to MC and MP, respectively.

At the genus level, the top 10 soil bacterial communities by relative abundance in each treatment were mainly composed of JG30-KF-AS9, *Acidothermus*, *Sphingomonas*, *Candidatus_Udaeobacter*, *Chujaibacter*, *Acidibacter*, *Chloroplast*, *Streptomyces*, WD2101_soil_group, and *Bryobacter*. Compared with the monoculture treatments, MC and MP, all intercropping treatments increased the relative abundance of *Candidatus_Udaeobacter* by 65.12–151.16% and 166.25–305.00%, respectively, with both IA and IB reaching significant levels (*p* < 0.05), and the increase in IB being the most significant. Conversely, intercropping treatments significantly reduced the relative abundance of *Acidothermus* and *Chujaibacter*, with the greatest decrease observed in treatment IA. Furthermore, compared with the monoculture of MC, intercropping treatments also significantly reduced the relative abundance of *Chloroplast*.

#### 3.4.3. Effects of Intercropping on the Alpha Diversity of Bacterial Communities

Based on the results of the bacterial alpha diversity analysis in Table 4, the ACE index for all intercropping treatments were higher than those for the monoculture treatments, MC and MP. However, the differences between the high-density intercropping treatment (IA) and the medium-density intercropping treatment (IB) were not statistically significant, indicating that intercropping helps enhance the species richness of bacterial communities. In terms of diversity, the Shannon index for all intercropping treatments was significantly higher than that of the monoculture treatments, while the Simpson index, although higher than that of the monoculture treatments, did not reach the level of statistical significance. This indicates that cassava–chili intercropping helps to increase the diversity of the bacterial community.

#### 3.4.4. Effects of Intercropping on the Beta Diversity of Bacterial Communities

**Figure 6:**
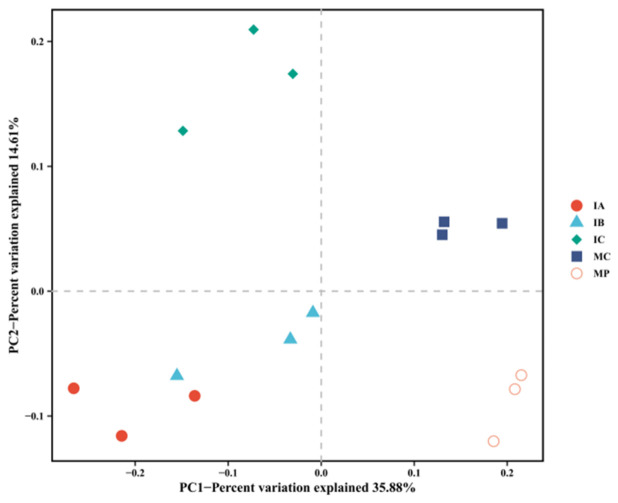
PCoA plot of beta diversity analysis of soil bacterial communities.

As shown in Figure 6, PCoA (principal coordinates analysis) of the soil bacterial communities in each treatment was conducted based on Bray–Curtis distance. The samples were clearly clustered and separated, and the overall model test was significant (R^2^ = 0.679, *p* = 0.001). PC1 and PC2 explained 35.88% and 14.61% of the total variance, respectively, with a cumulative explanation rate of 50.49%, which effectively reflects the overall pattern of differences among the samples. Tests of dispersion indicated no significant differences in dispersion among the groups (*p* > 0.05), and the PERMANOVA results were reliable. Based on the distribution of samples across treatments, distinct clustering and separation trends were observed between different treatments. Samples from the intercropping treatment groups showed distinct separation from the monoculture MC and MP groups, indicating that intercropping significantly altered the structure of the soil bacterial community. Furthermore, across the intercropping treatments, as intercropping density decreased, sample points on the PCoA plot exhibited a trend of shifting upward along the PC2 axis, suggesting that intercropping density may exert a gradient-like influence on the soil bacterial community by altering the intensity of interspecific competition and other factors.

#### 3.4.5. Redundancy Analysis of Bacterial Community Composition in Soil Environments

**Figure 7:**
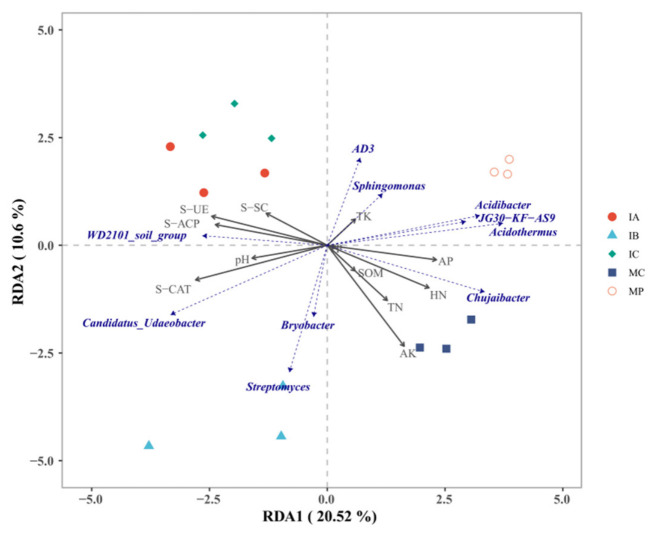
Redundancy analysis of the composition of soil bacterial communities in relation to soil environment. pH, soil Acidity and Alkalinity; TN, total nitrogen; TK, total potassium; HN, alkaline-hydrolyzable nitrogen; AP, available phosphorus; AK, available potassium; SOM, soil organic matter; S-CAT, catalase activity; S-UE, urease activity; S-SC; sucrase activity; S-ACP, acid phosphatase activity.

To investigate the effects of the soil environment on the composition of soil bacterial communities, we conducted a redundancy analysis (RDA). As shown in Figure 7, *Candidatus_Udaeobacter* was positively correlated with S-SC, S-UE, S-ACP, S-CAT, and pH; *Acidibacter*, JG30-KF-AS9, *Chujaibacter*, and *Acidothermus* were positively correlated with TK, AK, AP, HN, TN, and SOM; *Bryobacter* and *Streptomyces* were negatively correlated with TK; and *Sphingomonas* was positively correlated with TK.

## 4. Discussion

### 4.1. Effects of Intercropping on Crop Agronomic Traits

In this study, crop yields in cassava−chili intercropping systems exhibited significant density dependence. Cassava yields were highest in the low-density intercropping treatment (IC); chili yields, however, were highest in the monoculture treatment and were significantly higher than those in treatments IA and IC. This difference stems from the balance between interspecific competition and density compensation effects. Under high−density intercropping, cassava, leveraging its root system advantage, occupies a larger share of nutrients and water within the limited root zone, giving it a competitive advantage [32,33]. As the intercropping density of chili decreases, competition weakens, the inhibitory effect on cassava gradually diminishes, and yield recovers [34]. In medium−density intercropping of IB, chili may have mitigated yield losses per unit area through a population compensation effect, thereby maintaining their yield at higher intercropping densities [35]. For intercropping systems, the yield and economic benefits of all intercropping treatments were significantly higher than those of monoculture, which is consistent with the findings of Fu et al. [36].

In terms of quality, the intercropping treatment in this study significantly increased the starch and reducing sugar content of cassava. This may be attributed to the fact that competitive pressure from chilis during the symbiotic period activated storage organ demand signals: under low−density IC treatment, moderate competitive pressure promoted the expansion of storage organs, which subsequently sent feedback signals to the source leaves to enhance photosynthetic capacity; after chili harvest, the previously enhanced photosynthetic performance persisted, enabling cassava leaves to assimilate carbon at a higher net photosynthetic rate under full light, with photosynthetic products continuously transported to the tubers, thereby promoting starch accumulation [37,38]. In high-density intercropping systems, however, competition during the symbiotic phase is intense, shading is more severe, and enzymes involved in starch synthesis are inhibited, resulting in the accumulation of photosynthetic products in the tubers primarily in the form of reducing sugars [39]. The vitamin C and reducing sugar contents in chili were lowest in the high-density IA treatment. This may be due to the fact that, under high-density intercropping, interspecific competition between cassava and chilis, as well as intraspecific competition within the chili population, led to insufficient light within the population, resulting in a decrease in total photosynthetic products and a decline in fruit quality. In contrast, the moderate and low-density intercropping treatments provided moderate shading, which lowered fruit surface temperature and reduced photooxidative stress, thereby promoting the formation of vitamin C and reducing sugars [40,41].

### 4.2. Effects of Intercropping on Soil Biochemical Properties

Soil serves as a vital foundation for crop growth and ecosystem functioning, while crop intercropping can exert multidimensional effects on soil chemical fertility and microbial communities through niche complementarity and synergistic resource utilization among species. In this study, the regulation of soil physicochemical properties by intercropping exhibited distinct selectivity, primarily affecting the content of readily available nutrients in the soil. Specifically, the pH values of all intercropping treatments were significantly higher than those of chili monoculture, and the pH values of both IB and IC were also higher than those of cassava monoculture. In particular, low- to medium-density intercropping increased soil pH, which is consistent with the findings from studies on cassava−soybean intercropping [19], indicating that cassava−chili intercropping systems can mitigate soil acidification through root exudates and other mechanisms. However, Chen et al. [42] found that intercropping reduced pH in chili−maize systems, indicating that different crop combinations regulate soil pH in opposite directions. This may be related to differences in the types and quantities of organic acids secreted by the root systems of each crop. AN content was highest in the chili monoculture and significantly lower in the intercropping treatments, a finding which differs from the findings reported by Zhang et al. [43] that intercropping significantly increased AN content. This discrepancy may stem from cassava, as a high−biomass crop, competing intensely for nitrogen uptake. Furthermore, since both crops are non-legumes, they require more nitrogen for growth. Under intercropping conditions, the combined nitrogen consumption by both crops ultimately leads to reduced residual nitrogen, whereas in the chili monoculture, due to moderate planting density, and the absence of excessive inter-species competition, resulting in less loss of AN and allowing for its accumulation [44,45]. The content of available potassium was significantly lower in the intercropping treatment than in the sole-cropped cassava treatment, possibly because cassava’s well−developed root system has a strong capacity for potassium absorption and accumulation; after intercropping, the competitive uptake by chilis led to a reduction in residual soil potassium [32]. However, the available potassium content in the IB treatment was significantly higher than that in the chili monoculture, indicating that root interactions under medium-density intercropping may activate soil mineral potassium through root exudates, thereby promoting the conversion of non−exchangeable potassium to available potassium [46]. The available phosphorus content in intercropped systems is relatively low, which may be related to the efficient phosphorus uptake by cassava’s well-developed root system and the joint depletion of phosphorus reserves by both crops under intercropping conditions.

The overall enhancing effect of intercropping on soil enzyme activity was fully demonstrated in this study; the activities of soil catalase and acid phosphatase in all intercropping treatments were significantly higher than those in monoculture, which is consistent with the findings of Cui et al. [47] in a maize−soybean intercropping experiment. Farooq et al. [48] noted that in peanut-tea intercropping, increased soil enzyme activity enhanced the reaction rates of organic matter decomposition and nutrient release. The increase in acid phosphatase activity observed in this study may be attributed to a deficiency in available phosphorus caused by its joint consumption by both crops in the intercropping system. Plant roots and soil microorganisms responded by secreting more acid phosphatase to enhance the mineralization of organic phosphorus, thereby meeting the crops’ phosphorus requirements. The significant increase in catalase activity suggests that intercropping may have generated excess reactive oxygen species through root respiration and metabolic processes, with hydrogen peroxide being one of the main components. To mitigate the harmful effects of oxidative stress, microbial communities interact synergistically to promote an increase in soil catalase activity. Urease primarily originates from soil microorganisms such as bacteria and fungi, as well as plant roots. In this study, urease activity was higher in intercropping than in monocropping. This may be due, on the one hand, to increased root exudates promoting urease production, and on the other hand, to changes in the composition of the soil bacterial community driving the enhancement of urease activity. Similar results have been reported in cassava/peanut intercropping systems, where urease activity was significantly higher than in monoculture [18]. Sucrase activity was higher in all intercropping treatments than in the cassava monoculture, but lower in the high−density intercropping treatment compared to the chili monoculture. This may result from the interplay between the promotion of rhizosphere interactions and the suppression of competitive stress between the two crops under different intercropping densities. Overall, intercropping enhanced soil enzyme activity and improved the soil biochemical environment.

### 4.3. Effects of Intercropping on Soil Microbial Community Structure

Bacteria constitute the most abundant and active microbial group in soil, and numerous studies have shown that crop intercropping alters the structure of soil bacterial communities. In this study, intercropping significantly increased the richness and diversity of bacterial communities, consistent with the findings of Zhan et al. [49]. At the phylum level, the Acidobacteria phylum showed the greatest increase under high-density intercropping, likely because intense interspecific competition in high−density intercropping induced cassava roots to secrete more organic carbon, providing a carbon source for oligotrophic bacteria such as those in the Acidobacteria phylum [50]. The most significant increase in the phylum Verrucomicrobia was observed with medium-density intercropping, which is consistent with the results observed in the Chinese ginkgo intercropping system [51], this may be due to the fact that an appropriate intercropping density increases soil pH and total nitrogen content, thereby promoting the proliferation of the Verrucomicrobia phylum. The decline in the relative abundances of the Actinobacteria and Proteobacteria phyla in the cassava−chili intercropping system may be attributed to the absorption of readily available nutrients by the crops under intercropping conditions, which leads to reduced soil nutrient residues and changes in the composition of root exudates, thereby resulting in the selective enrichment of different phyla. In the intercropping system, the relative abundances of the Actinobacteria and Proteobacteria phyla—which are predominantly eutrophic—decreased, while those of the oligotrophic phyla Acidobacteria, Micrococcales, and Aeromonadales increased. This indicates that the soil bacterial community underwent functional reorganization to adapt to interspecific competition under the intercropping system. At the genus level, *Candidatus_Udaeobacter* is a widely distributed but difficult-to-culture genus in soil. In recent years, its important ecological functions in the rhizosphere microbiome have gradually been recognized. In studies on maize-peanut intercropping, it was found that *Candidatus_Udaeobacter* may be involved in soil antioxidant processes and organic matter decomposition [52], redundancy analysis in this study also confirmed that *Candidatus_Udaeobacter* is positively correlated with hydrogen peroxidase and pH. The enrichment of this bacterial genus in intercropping systems may be due to increased competition among the root systems of the two crops, which accelerates the decomposition of soil microorganisms, providing the bacteria with an abundant supply of metabolic substrates and reducing the energy required to synthesize these essential biomolecules on their own, thereby giving them a survival advantage in the soil environment [53]. In intercropping systems, this may be due to an increase in soil pH and changes in readily available nutrients, which disrupt the acidic environment that was originally conducive to *Acidothermus* growth; furthermore, intensified competition among crops and competition for resources within the soil bacterial community may have further limited their growth space, thereby leading to a decrease in their relative abundance [54]. *Chujaibacter* is classified as one of the representative groups of beneficial bacteria in soil; an increase in its abundance is associated with enhanced plant growth and the suppression of pathogens [55]. Studies have shown that intercropping increases the relative abundance of *Chujaibacter*, whereas in this study it decreased; this discrepancy may be related to the intercropping crop combination and intercropping density. In experiments involving the intercropping of Ophiopogon japonicus in tea gardens, Ophiopogon japonicus, being a perennial herb, exhibits weak competition with tea plants, which is conducive to the enrichment of *Chujaibacter* [56]. In this experiment, cassava, as a high-biomass crop, competed intensely with chilis; particularly in the high-density intercropping treatment, this intense interspecific competition may have altered the rhizosphere microenvironment, thereby inhibiting the accumulation of *Chujaibacter*.

## 5. Conclusions

This study investigated the effects of cassava−chili intercropping on crop yield and quality, soil biochemical properties, and soil bacterial community structure. The results confirmed that soil environments differed across various intercropping densities, and that low- to medium-density intercropping systems had beneficial effects on improving soil nutrient levels and enzyme activity, as well as reshaping the composition of soil bacterial communities.

## Figures and Tables

**Table 1:** Crop yield under different cropping systems.

Samples	Cassava (kg/ha)	Chili (kg/ha)	Total Yield (kg/ha)	Total Economic Benefit (RMB/ha)
IA	32,780.17 ± 662.10 b	12,586.24 ± 597.43 b	45,366.41 ± 1206.98 b	49,757.61 ± 1632.95 b
IB	33,554.91 ± 2012.84 b	13,265.62 ± 581.16 b	46,820.53 ± 2566.44 b	51,697.42 ± 2631.73 ab
IC	38,854.94 ± 933.78 a	12,714.38 ± 745.87 b	51,569.32 ± 1459.03 a	54,569.96 ± 1940.76 a
MC	34,088.50 ± 1359.51 b		34,088.50 ± 1359.51 c	25,566.38 ± 1019.64 c
MP		14,390.09 ± 752.38 a	14,390.09 ± 752.38 d	28,780.18 ± 1504.75 c

Values are presented as mean ± standard deviation. Different letters following the values within the same parameter indicate statistically significant differences (*p* < 0.05). Each treatment included three biological replicates (n = 3).

**Table 2:** Quality under different cropping systems.

Samples	Cassava		Chili	
	Starch (mg/g)	Reducing Sugars (mg/g)	Vc (mg/g)	Reducing Sugars (mg/g)
IA	286.358 ± 7.295 ab	2.410 ± 0.088 a	1.15 ± 0.07 b	13.27 ± 0.58 c
IB	274.183 ± 6.636 b	2.311 ± 0.117 a	1.30 ± 0.06 ab	22.10 ± 3.76 a
IC	302.248 ± 6.767 a	1.635 ± 0.058 b	1.38 ± 0.16 a	18.81 ± 1.08 ab
MC	253.281 ± 14.494 c	1.364 ± 0.170 c		
MP			1.35 ± 0.06 a	15.40 ± 1.34 bc

Values are presented as mean ± standard deviation. Different letters following the values within the same parameter indicate statistically significant differences (*p* < 0.05). Each treatment included three biological replicates (n = 3).

**Table 3:** Analysis of differences in composition at the phyla and genus levels.

	Treatment	IA	IB	IC	MC	MP
Phylum level	Actinobacteriota	24.56 ± 1.37 bc	26.96 ± 1.15 b	24.37 ± 1.25 c	31.80 ± 0.55 a	31.46 ± 2.05 a
	Proteobacteria	19.61 ± 0.39 b	19.96 ± 0.28 b	21.08 ± 1.43 ab	21.98 ± 1.06 a	22.7 ± 1.03 a
	Chloroflexi	15.94 ± 1.05 b	15.94 ± 0.50 b	16.19 ± 0.69 b	13.46 ± 0.80 c	18.09 ± 1.52 a
	Acidobacteriota	12.75 ± 1.53 a	9.62 ± 0.38 bc	10.71 ± 1.46 b	7.92 ± 1.14 cd	7.47 ± 0.24 d
	Planctomycetota	6.11 ± 0.09 ab	6.44 ± 0.25 a	5.33 ± 0.44 bc	5.21 ± 0.31 c	5.16 ± 0.80 c
	Verrucomicrobiota	4.65 ± 0.20 a	4.78 ± 0.88 a	3.90 ± 0.67 a	2.53 ± 0.40 b	2.09 ± 0.25 b
	Bacteroidota	1.81 ± 0.67 b	2.68 ± 0.59 b	5.37 ± 2.62 a	1.99 ± 1.05 b	1.49 ± 0.34 b
	Gemmatimonadota	2.68 ± 0.14 a	2.35 ± 0.12 ab	2.69 ± 0.60 a	1.55 ± 0.24 c	2.02 ± 0.09 bc
	Patescibacteria	2.05 ± 0.59 b	1.95 ± 0.55 b	1.73 ± 0.17 b	3.35 ± 0.91 a	2.04 ± 0.41 b
	Firmicutes	1.74 ± 0.1 cd	2.01 ± 0.28 bc	1.51 ± 0.25 d	2.46 ± 0.38 b	3.05 ± 0.13 a
Genus level	JG30-KF-AS9	4.88 ± 0.98 b	5.75 ± 0.32 b	5.98 ± 0.57 b	6.56 ± 0.81 b	9.05 ± 1.95 a
	*Acidothermus*	2.42 ± 0.54 b	2.46 ± 0.49 b	2.65 ± 0.14 b	3.54 ± 0.17 a	4.17 ± 0.21 a
	*Sphingomonas*	2.65 ± 0.46 a	2.26 ± 0.22 a	2.35 ± 0.39 a	2.34 ± 0.64 a	3.02 ± 0.38 a
	*Candidatus_Udaeobacter*	2.35 ± 0.08 b	3.24 ± 0.92 a	2.13 ± 0.33 bc	1.29 ± 0.30 cd	0.8 ± 0.27 d
	*Chloroplast*	1.5 ± 0.74 b	1.36 ± 0.35 b	1.12 ± 0.37 b	4.15 ± 3.09 a	0.91 ± 0.16 b
	*Acidibacter*	1.24 ± 0.30 b	1.48 ± 0.18 b	1.38 ± 0.27 b	1.71 ± 0.22 b	3.05 ± 0.27 a
	*Streptomyces*	1.34 ± 0.86 b	3.2 ± 0.30 a	0.62 ± 0.11 b	1.39 ± 0.09 b	1.18 ± 0.24 b
	*Chujaibacter*	0.26 ± 0.06 d	1.61 ± 0.95 bc	0.73 ± 0.18 cd	2.32 ± 0.29 ab	2.81 ± 0.71 a
	*Bryobacter*	1.09 ± 0.09 b	1.4 ± 0.05 a	1.21 ± 0.13 ab	1.17 ± 0.17 ab	1.22 ± 0.26 ab
	WD2101_soil_group	1.36 ± 0.19 a	1.28 ± 0.08 ab	1.20 ± 0.10 abc	0.86 ± 0.29 c	0.93 ± 0.2 bc

Values are presented as mean ± standard deviation. Different letters following the values within the same parameter indicate statistically significant differences (*p* < 0.05). Each treatment included three biological replicates (n = 3).

**Table 4:** The effect of intercropping on the Alpha Diversity Index of soil bacterial communities.

Treatment	Richness	Diversity	
	ACE Index	Shannon Index	Simpson Index
IA	5483.67 ± 100.16 a	10.52 ± 0.09 a	0.998 ± 0.000 a
IB	5596.67 ± 278.44 a	10.45 ± 0.12 a	0.998 ± 0.001 ab
IC	4553.67 ± 270.60 b	10.34 ± 0.23 a	0.997 ± 0.001 abc
MC	4168.67 ± 161.30 b	9.92 ± 0.22 b	0.996 ± 0.001 bc
MP	3179.67 ± 286.99 c	9.45 ± 0.02 c	0.996 ± 0.000 c

Values are presented as mean ± standard deviation. Different letters following the values within the same parameter indicate statistically significant differences (*p* < 0.05). Each treatment included three biological replicates (n = 3).

## Data Availability

The data presented in this study are available upon request from the corresponding author.

## References

[1] Amelework A.B., Bairu M.W. (2022). Advances in Genetic Analysis and Breeding of Cassava (*Manihot esculenta* Crantz): A Review. Plants.

[2] Lambebo T., Deme T., Geleta M. (2025). Nutritional enhancement of cassava through processing: Implications for sustainable food systems. Front. Sustain. Food Syst..

[3] Fu H., Qu Y., Pan Y. (2018). Efficiency of Cassava Production in China: Empirical Analysis of Field Surveys from Six Provinces. Appl. Sci..

[4] Alonso-Villegas R., González-Amaro R.M., Figueroa-Hernández C.Y., Rodríguez-Buenfil I.M. (2023). The Genus *Capsicum*: A Review of Bioactive Properties of Its Polyphenolic and Capsaicinoid Composition. Molecules.

[5] Mohd A.M., Khalid N.I., Wondi M.H., Haris N.I.N., Azman P.N.M.A. (2025). Exploring the nutritional values, volatile compounds, health benefits, and potential food products of chili (*Capsicum annuum*): A comprehensive review. Food Chem..

[6] Liu Z.F., Huang J., Wei Y.X., Sun H.R. (2016). Effect of Yield Performance and Economic Returns in Different Cassava/Peanut Intercropping System. Chin. J. Trop. Crops.

[7] Toker P., Canci H., Turhan I., Isci A., Scherzinger M., Kordrostami M., Yol E. (2024). The advantages of intercropping to improve productivity in food and forage production—A review. Plant Prod. Sci..

[8] Li C.T., Stomph D., Makowski H., Li C., Zhang F., Zhang W. (2023). The productive performance of intercropping. Proc. Natl. Acad. Sci. USA.

[9] Tang Y.R., Qiu Y.R., Li X., Qin H.Y., Wang J., Zhang S.J., Han Y.C., Feng L., Wang G.P., Yang B.F. (2024). Increased overyielding probability and yield stability from a 5-year cotton-based intercropping. Eur. J. Agron..

[10] Akchaya K., Parasuraman P., Pandian K., Vijayakumar S., Thirukumaran K., Mustaffa M., Rajpoot S.K., Choudhary A.K. (2025). Boosting resource use efficiency, soil fertility, food security, ecosystem services, and climate resilience with legume intercropping: A review. Front. Sustain. Food Syst..

[11] Carton N., Naudin C., Piva G., Corre-Hellou G. (2020). Intercropping Winter Lupin and Triticale Increases Weed Suppression and Total Yield. Agriculture.

[12] De Long J.R., van Malland F., de Buck A., van den Berg M. (2023). Wheat and faba bean intercropping and cultivar impacts on morphology, disease, and yield. Agron. J..

[13] Wen B., Zhang X., Ren S., Duan Y., Zhang Y., Zhu X., Wang Y., Ma Y., Fang W. (2020). Characteristics of soil nutrients, heavy metals and tea quality in different intercropping patterns. Agrofor. Syst..

[14] Zhang W.J., Wang P., Chen X.X., Feng H., Zhu L.X. (2018). Effects of *Platycodon grandiflorum*/chili intercropping on root growth, yield and quality of *Platycodon grandiflorum*. China J. Chin. Mater. Medica.

[15] Duchene O., Vian J.V., Celette F. (2017). Intercropping with legume for agroecological cropping systems: Complementarity and facilitation processes and the importance of soil microorganisms. A review. Agric. Ecosyst. Environ..

[16] Du H., Lin Y., Qi M., Qu P., Xu Z., Lin R., Xie C., Xiao T., Dong S., Wang B. (2026). Intercropping Amomum villosum enhances soil stratification, nutrient complementarity, and microbial communities in rubber plantations. Front. Microbiol..

[17] Zhang M.M., Wang N., Hu Y.B., Sun G.Y. (2018). Changes in soil physicochemical properties and soil bacterial community in mulberry (*Morus alba* L.)/alfalfa (*Medicago sativa* L.) intercropping system. MicrobiologyOpen.

[18] Tang X.M., Zhong R.C., Jiang J., He L.Q., Huang Z.P., Shi G.Y., Wu H.N., Liu J., Xiong F.Q., Han Z.Q. (2020). Cassava/peanut intercropping improves soil quality via rhizospheric microbes increased available nitrogen contents. BMC Biotechnol..

[19] Chen H., Ruan L., Cao S., He W., Yang H., Liang Z., Li H., Wei W., Huang Z., Lan X. (2025). Cassava–soybean intercropping alleviates continuous cassava cropping obstacles by improving its rhizosphere microecology. Front. Microbiol..

[20] He C., Zhou B., Wang H., Wei Y., Huang J. (2023). A first-year maize/cassava relay intercropping system improves soil nutrients and changes the soil microbial community in the symbiotic period. Front. Microbiol..

[21] Abirami V., Velmurugan M., Thangamani C., Natarajan S.K., Davamani V., Indu R.C., Venkatachalam S.R. (2025). Cassava intercropping systems for enhanced land productivity and farmer livelihoods: A review. Plant Sci. Today.

[22] Olasantan F.O., Salau A.W., Onuh E.E. (2007). Influence of cassava (manihot esculenta) intercrop on growth and fruit yields of pepper (*Capsicum* spp.). Exp. Agric..

[23] Philippot L., Chenu C., Kappler A., Rillig M.C., Fierer N. (2024). The interplay between microbial communities and soil properties. Nat. Rev. Microbiol..

[24] Kiprotich K., Muema E., Wekesa C., Ndombi T., Muoma J., Omayio D., Ochieno D., Motsi H., Mncedi S., Tarus J. (2025). Unveiling the roles, mechanisms and prospects of soil microbial communities in sustainable agriculture. Discov. Soil.

[25] Wang J., Bai C., Tian Y., Bao J., Liu J. (2026). Intercropping reshapes soil stress resistance and growth promotion capabilities through rhizosphere exudates in conjunction with the microbiome. Front Microbiol..

[26] Mao J., Huang Y.J., Song J., Zhao X.F., Tang W. (2021). Application of Decision Unit-Multi Increment Sampling in synchronized soil and crop sampling in heavy metal-contaminated farmland. J. Agro-Environ. Sci..

[27] Bao S. (2000). Soil Chemical Analysis.

[28] Kandeler E., Gerber H. (1988). Short-term assay of soil urease activity using colorimetric determination of ammonium. Biol. Fert. Soils.

[29] Li Z.Y., Zheng L. (2016). Soil Sucrase: Detection Conditions Based on DNS Colorimetric. Chin. Agric. Sci. Bull..

[30] Velez A., Rojas L., Salcedo M., Valderrama C. (2024). Methods to determine enzymatic activity in contaminated soils. South Sustain..

[31] Tabatabai M.A., Bremner J.M. (1969). Use of *p*-nitrophenyl phosphate for assay of soil phosphatase activity. Soil Biol. Biochem..

[32] Qi Z.P. (2000). Effect of Cassava-Arachis pentoi Intercropping System on Root Space Variety and Soil Quality. J. South China Agric. Univ..

[33] Postma J.A., Lynch J.P. (2012). Complementarity in root architecture for nutrient uptake in ancient maize/bean and maize/bean/squash polycultures. Ann. Bot..

[34] Wang Y., Han X., Zhao X., Zhang Y., Qi B., Li L. (2024). Grain yield and interspecific competition in an oat-common vetch intercropping system at varying sowing density. Front. Plant Sci..

[35] Wang J., Li S.J., Chen J.F., Xie H.H., Chen M., Zhang H.L., Sheng H., Song Y. (2025). Effects of Intercropping Cassava on the Growth, Yield, Quality, and Soil Enzyme Activity of Fresh Soybeans. J. Fujian Agric..

[36] Fu Z., Chen P., Luo K., Lin P., Li Y., Pu T., Li Y., Wu Y., Wang X., Yang W. (2025). Relay strip intercropping of soybeans and maize achieves high net ecosystem economic benefits by boosting land output and alleviating greenhouse gas emissions. J. Sci. Food Agric..

[37] Matthew J.P., Christine H.F. (2001). Sink regulation of photosynthesis. J. Exp. Bot..

[38] Smith M.R., Rao I.M., Merchant A. (2018). Source-Sink Relationships in Crop Plants and Their Influence on Yield Development and Nutritional Quality. Front. Plant Sci..

[39] Ding Z., Zhang Y., Xiao Y., Liu F., Wang M., Zhu X., Liu P., Sun Q., Wang W., Peng M. (2016). Transcriptome response of cassava leaves under natural shade. Sci. Rep..

[40] Caruso G., Cozzolino E., Cuciniello A., Maiello R., Cenvinzo V., Giordano M., De Pascale S., Rouphael Y. (2020). Yield and quality of greenhouse organic pepper as affected by shading net in Mediterranean area. Acta Hortic..

[41] Kabir M.Y., Bautista J., Dutta B., Nambeesan S.U., Díaz-pérez J.C. (2022). Mineral nutrients, physiological disorders, postharvest water loss, and PR gene expression in bell pepper (*Capsicum annuum* L.) fruit under shade nets. J. Hortic. Res..

[42] Chen Z., Wang W., Chen L., Zhang P., Liu Z.H., Yang X.K., Shao J.L., Ding Y., Mi Y.H. (2024). Effects of pepper–maize intercropping on the physicochemical properties, microbial communities, and metabolites of rhizosphere and bulk soils. Environ. Microbiome.

[43] Zhang W., Zhao Y., Li G., Shen L., Wei W., Li Z., Tuerti T., Zhang W. (2025). The Effects of Maize–Soybean and Maize–Peanut Intercropping on the Spatiotemporal Distribution of Soil Nutrients and Crop Growth. Agronomy.

[44] Obi E.A., Agele S.O., Aiyelari O.P., Adejoro S.A., Agbona A.I. (2022). Nutrient uptake and use efficiencies of strip intercropped cassava, maize and chili as affected by fertilizer type and age of oil palm fields in an oil palm-based intercropping system. J. Soil Sci. Environ. Manag..

[45] Rahman M.K.U., Saati-Santamaría Z., García-Fraile P. (2025). Intercropping of non-leguminous crops improves soil biochemistry and crop productivity: A meta-analysis. New Phytol..

[46] Zhou D.Y., Sun Y.X., Su H.J., Li S.X., Dong Q.Q., Zhang Y.C., Zhang H., Wang J., Wang X.G., Yu H.Q. (2025). Effects of Root Interaction Intensity on the Activation and Uptake of Potassium in Soil under Maize/Peanut Intercropping. J. Shenyang Agric. Univ..

[47] Cui J., Li S., Baoyin B., Feng Y., Guo D., Zhang L., Gu Y. (2024). Maize/Soybean Intercropping with Straw Return Increases Crop Yield by Influencing the Biological Characteristics of Soil. Microorganisms.

[48] Farooq T.H., Kumar U., Mo J., Shakoor A., Wang J., Rashid M.H.U., Tufail M.A., Chen X., Yan W. (2021). Intercropping of Peanut-Tea Enhances Soil Enzymatic Activity and Soil Nutrient Status at Different Soil Profiles in Subtropical Southern China. Plants.

[49] Zhan X., Shu Y., Guo L., Liu X., Zhao Q., Li Y., Yong T., Yang W. (2025). Response of soil microbial community diversity and structure to soybean-based intercropping and its effects on yield. Front. Microbiol..

[50] Liu Z., Nan Z., Lin S., Meng W., Xie L., Yu H., Zhang Z., Wan S. (2024). Peanut-based intercropping systems altered soil bacterial communities, potential functions, and crop yield. PeerJ.

[51] Peng F.D., Wei F., Wang H.L., Jian H.T., Xu X.N. (2022). Chinese Torreya agroforestry alters the rhizosphere soil bacterial communities and potential functions. Appl. Soil Ecol..

[52] Zhao X., Dong Q., Han Y., Zhang K., Shi X., Yang X., Yuan Y., Zhou D., Wang K., Wang X. (2022). Maize/peanut intercropping improves nutrient uptake of side-row maize and system microbial community diversity. BMC Microbiol..

[53] Willms I.M., Rudolph A.Y., Göschel I., Bolz S.H., Schneider D., Penone C., Poehlein A., Nacke H. (2020). Globally Abundant “*Candidatus Udaeobacter*” Benefits from Release of Antibiotics in Soil and Potentially Performs Trace Gas Scavenging. mSphere.

[54] Zhang J.T., Chang J., Yang S.P., Yang X.H., Wang K.L., Ge X. (2023). Study on soil microbial community of pecan-wheat intercropping system. J. China Agric. Univ..

[55] Freitas A.S., Zagatto L.F.G., Rocha G.S., Muchalak F., Martins G.L., Silva-Zagatto S.S., Hanada R.E., Muniz A.W., Tsai S.M. (2025). Harnessing the synergy of Urochloa brizantha and Amazonian Dark Earth microbiomes for enhanced pasture recovery. BMC Microbiol..

[56] Li Q.S., Lei W.X., Liu J.X., Zhu J.W., Shi L.S., Cai P.M., Zhang J.M., Jia X.L. (2021). Effects of intercropping Ophiopogon japonicus into tea plantation on its soil physicochemical properties and microbial community structure. J. South. Agric..

